# High intensity focused ultrasound for the treatment of solid tumors: a pilot study in canine cancer patients

**DOI:** 10.1080/02656736.2022.2097323

**Published:** 2022

**Authors:** Jennifer Carroll, Sheryl Coutermarsh-Ott, Shawna L. Klahn, Joanne Tuohy, Sabrina L. Barry, Irving C. Allen, Alayna N. Hay, Jeffrey Ruth, Nick Dervisis

**Affiliations:** a Department of Small Animal Clinical Sciences, Virginia-Maryland College of Veterinary Medicine, Blacksburg, VA, USA; b Department of Biomedical Sciences and Pathobiology, Virginia-Maryland College of Veterinary Medicine, Blacksburg, VA, USA; c Department of Basic Science Education, Virginia Tech Carilion School of Medicine, Roanoke, VA, USA; d Department of Internal Medicine, Virginia Tech Carilion School of Medicine, Roanoke, VA, USA; e ICTAS Center for Engineered Health, Virginia Tech, Blacksburg, VA, USA

**Keywords:** High-intensity focused ultrasound, thermal ablation, immunotherapy, Dog

## Abstract

**Purpose:**

To investigate the safety, feasibility, and outcomes of High-Intensity Focused Ultrasound (HIFU) for the treatment of solid tumors in a spontaneous canine cancer model.

**Methods:**

Dogs diagnosed with subcutaneous solid tumors were recruited, staged and pretreatment biopsies were obtained. A single HIFU treatment was delivered to result in partial tumor ablation using a commercially available HIFU unit. Tumors were resected 3–6 days post HIFU and samples obtained for histopathology and immunohistochemistry. Total RNA was isolated from paired pre and post treated FFPE tumor samples, and quantitative gene expression analysis was performed using the nCounter Canine IO Panel.

**Results:**

A total of 20 dogs diagnosed with solid tumors were recruited and treated in the study. Tumors treated included Soft Tissue Sarcoma (*n* = 15), Mast Cell Tumor (*n* = 3), Osteosarcoma (*n* = 1), and Thyroid Carcinoma (*n* = 1). HIFU was well tolerated with only 1 dog experiencing a clinically significant adverse event. Pathology confirmed the presence of complete tissue ablation at the HIFU targeted site and immunohistochemistry indicated immune cell infiltration at the treated/untreated tumor border. Quantitative gene expression analysis indicated that 28 genes associated with T-cell activation were differentially expressed post-HIFU.

**Conclusions:**

HIFU appears to be safe and feasible for the treatment of subcutaneous canine solid tumors, resulting in ablation of the targeted tissue. HIFU induced immunostimulatory changes, highlighting the canine cancer patient as an attractive model for studying the effects of focal ablation therapies on the tumor microenvironment.

## Introduction

High Intensity Focused Ultrasound (HIFU) is a noninvasive ablation technique that has been used to effectively treat a variety of pathologies in people [1–9]. Treatment with HIFU results in a plethora of biological effects, dependent on the treatment parameters utilized [10,11]. There is growing interest in the use of ultrasound for alternative applications such as sonothrombolysis, blood brain barrier opening, sonoporation to enhance chemotherapeutic and gene delivery, treatment of neurodegenerative diseases, and immunomodulation, most of which has been evaluated using *in vitro* and *in vivo* models [12–19].

HIFU can also elicit an anti-tumor immune response. Preclinical studies using murine tumor models have indicated an increase in the number of activated dendritic cells (APCs) as evidenced by increased expression of MHC class II, CD80, and CD86, as well as increased levels of IL-12 and IFNγ in the tumor microenvironment post HIFU [20–22]. Zhong-lin et al. reported similar increases in CD80/86+ dendritic cells in breast cancer patients treated with HIFU when compared to controls managed with radical mastectomy [23]. Activated dendritic cells loaded with antigen from HIFU ablated tumors as well as adoptive transfer of HIFU activated cytotoxic T-lymphocytes have proven efficacious in preventing and/or delaying tumor regrowth following re-challenge [24,25].

To date the use of HIFU for the treatment of canine cancer is scarce and limited to small retrospective case series or reports [26–30]. Ryu et al. evaluated the use of HIFU for the treatment of solid tumors in canines and found that 5/10 dogs treated experienced relief of their clinical signs and 4/10 dogs had documented tumor regression of varying degrees. Side effects were mild consisting of erythema, superficial skin ulceration, and enteritis, all of which were self-limiting [27]. Another study investigated the use of sonodynamic therapy using an anti-cancer micelle and HIFU combination for the treatment of 4 different tumor histologies. Treatment resulted in improvement in patient pain scores and function [28]. Kopelman et al. used MRI guided Focused Ultrasound with thermometry for the treatment of a large hepatocellular adenoma in a dog over the course of 4 separate sessions. Finally, Ranjan et al., reported on a case of a large oral tumor treated with HIFU, resulting in tumor remission and proliferation of T-cells around the treated tumor [29].

Clinical trials involving pet dogs allow for the study of naturally arising tumors in immunocompetent hosts that share the same environmental risk factors as their owners. Additionally, a variety of canine cancers have been found to have similar histology, cytogenetic abnormalities, and oncogenic drivers as their human counterparts [31–35]. The purpose of this pilot study was to prospectively evaluate the safety, feasibility, and effectiveness of standardized HIFU for the treatment of canine solid tumors, and characterize the short-term intra-tumoral immune response to the treatment.

## Methods

### Study design

This study was approved by the Institutional Animal Care and Use Committee at Virginia Tech (IACUC # 17-231). Dogs with cytologically or histologically diagnosed subcutaneous solid tumors were recruited for the study through the Virginia-Maryland Veterinary Teaching Hospital and affiliated Collaborative Research Network between May 2018 and February 2020. Patient screening for enrollment included a complete blood count, serum chemistry, urinalysis, prothrombin time, partial thromboplastin time, computed tomography (CT) with contrast enhancement imaging of the thorax, abdomen, and tumor site, and needle core biopsy of the tumor. Dogs were eligible for inclusion based on the criteria listed in Table 1. Metastatic disease, as seen on cross-sectional imaging, was not considered a reason for exclusion if it was not expected to result in a survival of <6 weeks or jeopardize anesthetic safety. Written informed consent by the dog owners was obtained before study enrollment.

### HIFU treatment

The HIFU treatment was delivered using a commercially available unit (Echopulse, Theraclion, Malakoff, France). The unit consists of a visualization and treatment unit (VTU) suspended from a robotic arm attached to the electronic cabinet base. The cabinet includes a cooling unit circulating de-gassed water in a closed circuit between the cooling pouch and the balloon located on the VTU, allowing the ultrasound to be transmitted to the patient while simultaneously protecting the skin from thermal injury. The VTU includes a piezoelectric treatment transducer and imaging probe that allows for real-time monitoring of the target during treatment. The transducer is comprised of a single element operating at 3 MHz and has a focal length of 38 mm. The imaging probe is composed of a linear array of 128 elements that operates at frequencies between 5 and 10MHz.

All HIFU treatments were performed with the patients under general anesthesia. Additionally, patients with tumors localized on an extremity received a local nerve block. The HIFU treatment site was prepared using a combination of electrical clippers, razors, and waxing. Diagnostic ultrasound imaging was performed prior to treatment to obtain tumor measurements and identify critical structures. A combination of CT imaging as well as pretreatment and real time ultrasonographic examination was used to identify the tumor, skin, critical structures (blood vessels, nerves, bone), and tumor necrotic areas. A target volume, representing the volume within the tumor to be treated, was selected to be between 5 and 27 mm in depth. For all patients, the target volume represented only a portion of the total tumor volume. Given the large range in patient tumor size (<2cm^3^ to >10cm^3^), an attempt to treat a set percentage of the total tumor volume was not made. The target volume allowed for a 5 mm safety margin from the skin surface and 2 mm safety margin from large blood vessels and nerves, and was selected so that the total treatment time did not exceed 1 h. Cavitated portions of the tumor as well as regions containing macrocalcifications in the near field or tumor were excluded during treatment target selection. The target volume was divided into voxels that represent the volume treated with one pulse. The approximate height and width of each unitary treatment volume is 7.3 mm and 5 mm respectively, which equates to approximately 0.1 mL or 0.1 cm^3^. As each pulse followed by the required cooling time (dependent on target site depth and power) ranged between 40 and 50 s in duration, an approximate treatment time of 30–60 min would be expected to result in an ablation zone of 3–6cm^3^.

Patients were positioned in a manner that optimized VTU contact with the target tissue and coupling was achieved *via* ultrasound gel. Using the integrated treatment planning software, the skin, tumor, and sensitive structures were manually delineated by tracing their borders. After the intended tumor treatment volume, skin, and critical structures were determined in the sagittal and transverse planes for each slice, the planning software divided the lesion into evenly spaced treatment voxels. Predetermined safety margins determining the number and spacing of voxels in the treatment plan included a minimum of 5 mm distance from the skin surface and 2 mm from major vessels. The derrated power applied during the treatment was selected by firing a ranging pulse to determine the power needed to induce a hyperechoic mark [36]. The VTU was robotically driven to sequentially ablate the tissue represented by each voxel by applying ultrasound pulses for a period of 10 s, followed by a cooling phase of 30 s duration. The HIFU treatment was delivered in a single session, with the ablation volume planned to be smaller than the total tumor volume. Post treatment, dogs were recovered from anesthesia and released to their owners, with an Elizabethan-collar to prevent any self-trauma to the skin over the treated area. No anti-inflammatory or pain medications were dispensed.

### Tumor resection

The first 3 dogs in the study were hospitalized in our Intensive Care Unit (ICU) for monitoring starting from the HIFU treatment until surgery (4 days later). No significant adverse events requiring hospitalization were observed, thus the remainder of dogs were released to their owners post-HIFU until their surgical appointment, 4–6 days later. Tumor resection was performed using standard surgical techniques, depending on the anatomical location and surgeon preference. Dogs were recovered in the ICU and discharged 1–2 days post-surgery. Recheck examinations were performed 2 weeks after surgical removal, and every 3–4 months thereafter.

### Histopathology and immunohistochemistry

Pre-HIFU therapy tumor biopsy samples and post-HIFU resected tumor samples were processed within 20 min of excision. Resected tumor sections were evaluated by a board-certified pathologist (SCO) for definitive diagnosis, margin assessment, and identification of the ablated tissue. The treatment site and overlying skin were grossly evaluated prior to formalin fixation. Sections of the treated and untreated tumor were collected and microscopically evaluated. Sections of overlying skin were assessed microscopically when evidence of potential thermal injury was grossly visible. All sections for microscopy were routinely processed and stained with hematoxylin and eosin (H&E). Immunohistochemistry for CD3 (rabbit polyclonal, anti-human; A0452; Agilent/Dako, Santa Clara, California), IBA-1 (rabbit polyclonal, anti-human; NC9288364; Wako Chemicals, USA), and CD79a (mouse monoclonal, anti-human; sc-20064; Santa Cruz Biotechnology) was performed. All antibodies were validated and run on a Ventana Benchmark XP automated stainer (Roche Ventana, Oro Valley, Arizona) using the Discovery Universal secondary antibody (760-4205; Roche, Basel, Switzerland), ultraView Universal Alkaline Phosphatase Red Detection Kit (Roche), and hematoxylin counterstain. All antibodies were verified to work in canine tissue before use in research samples.

### Adverse event assessment

Potential damage to the skin overlying the treatment site was assessed clinically and on histopathology. Burn lesion classification was performed according to the grading scheme described by Wohlsein et al. [37]. Briefly, first degree burns are restricted to the epidermis. Superficial second-degree burns spare the basal layer of the epidermis that contacts the basement membrane while deep second-degree burns involve damage to the basal layer while sparing adnexal structures. Third-degree burns involve destruction of both the epidermis and dermis. Fourth-degree burns extend beyond the epidermis and dermis into the subcutaneous tissue and deeper tissue including muscle and bone [37]. For other adverse events, the Veterinary Cooperative Oncology Group-Common Terminology Criteria for Adverse Events following investigational therapy in dogs and cats was used [38].

### Gene expression analysis

Gene expression analysis was performed on tumor samples from dogs diagnosed with soft tissue sarcoma. Formalin-fixed, paraffin-embedded (FFPE) pre- and post-HIFU tumor tissue was used for total RNA isolation. Samples used included pretreatment biopsy specimens and post treatment surgical specimens procured 4–6 days following treatment. Sections of pretreated tumor and of the zone at the interface of ablated/nonablated tumor post-treatment with HIFU were selected for RNA extraction. A random number generator was used to determine the order in which samples were processed. We dissected the zone between the ablated tumor tissue and non-ablated tumor tissue (transition zone), from 20-μm thick slices of each post-treatment FFPE tissue sample and RNA extraction was performed using a Zymo kit according to the manufacturer’s directions (Zymo, Irvine, California). Quantification and qualification of RNA was performed using Nanodrop (NanoDrop One/OneC, Thermo Fisher Scientific).

RNA (100 ng) in 5 μL was hybridized with gene-specific reporter and capture probes (nCounter Canine IO panel, NanoString, 530 Fairview Ave N, Seattle, WA 98109) at 65 °C for 18 h and processed on the nCounter Prep station, and data were acquired using nCounter scanner. Profiled data were pre-processed, background was subtracted by using threshold counts of 20, normalization was performed with positive control and housekeeping genes using Rosalind software (rosalind.bio). Obtained values were Log2 transformed prior to identification of differentially expressed (DE) genes (*p* < .05) using heteroscedastic t tests per manufacturers’ recommendation. DEs were plotted using agglomerative clustering (Euclidean distance), and fold changes and p values were reported for each DE.

### Statistical analysis

Descriptive statistics were used to summarize the patient population, epidemiology, and tumor characteristics. Disease free survival (DFS) was defined as the time from HIFU treatment, until evidence of tumor recurrence or metastasis. Overall survival (OS) was defined as the time from HIFU treatment until patient death due to disease. Patient death was defined as due to disease unless the Medical Records indicated otherwise. Patients were censored if they were still alive at the time of data analysis, died due to other causes, or were lost to follow-up. The Kaplan–Meier survival analysis method was used to estimate DFS and OS curves following HIFU. All reported p-values were two-sided and *p*-values <.05 were considered statistically significant. Statistical analyses were performed with standard software (JMP Pro 16 Statistical Software, www.jmp.com).

## Results

### Canine patient characteristics

Twenty dogs were enrolled in the study, with 12 males and 8 females. Of the males and females, all but 1 from each group were castrated or spayed. The median age at presentation was 10.5 years (range:7–13yrs). The study population included a mixture of purebred and mixed breed dogs. The mean weight was 28.02 kg (±12.6). The tumors were located over the distal extremities in 15/20 dogs, the trunk in 3/20 dogs, the ventral cervical region in 1/20 dogs, and the tail base in 1/20 dogs. Tumor histologies represented in this study were: 15 soft tissue sarcomas, 1 osteosarcoma, 3 mast cell tumors, and 1 thyroid carcinoma (Table 2).

### Feasibility and safety of HIFU

**Figure 1.**
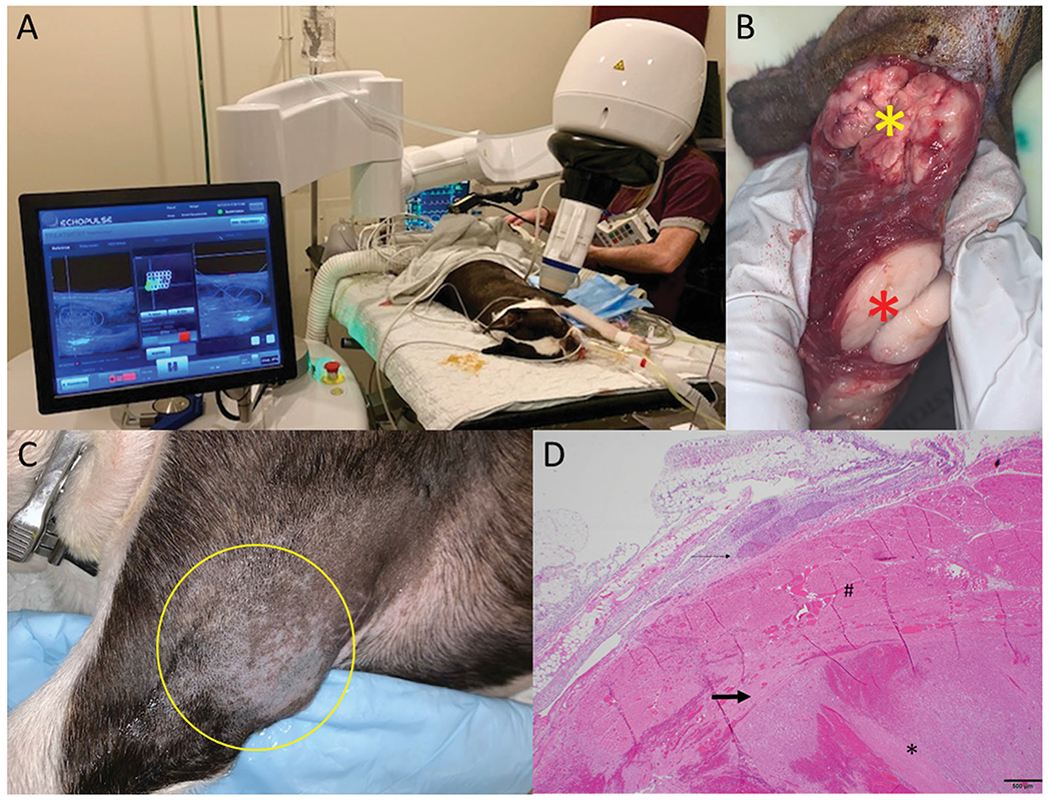
HIFU treatment of a dog diagnosed with 2 masses located on the proximal left leg, infiltrating the triceps muscle. Both masses were diagnosed as low grade liposarcoma. (A) Treatment setup. The dog is under inhalational general anesthesia in combination with local block of his left brachial nerve. The VTU is positioned over the targeted tumor, and the treatment is monitored in real time *via* ultrasonography. (B) Gross image of the resected tumors. The yellow star indicates the treated tumor, characterized by diffuse hemorrhage and tan color, and the red star indicates the untreated tumor, lacking hemorrhage and pale white color. (C) The tumor treatment site (yellow circle) 4 days post HIFU treatment with no visible evidence of heat damage. (D) Microscopy image of the treated tumor stained with standard H&E, demonstrating coagulative necrosis and haemorrhage.

Twenty dogs were treated with a single tumor targeted per dog. Eighteen dogs had their tumors treated and resected according to protocol, while two dogs had their tumors treated with HIFU and monitored over time, due to tumor anatomical localization restrictions. The mean tumor treatment duration ± SD was 26.5 ± 14.2 min, for an average 30 ± 17 treated sites per tumor. The mean delivered energy per tumor was 8 ± 5.2 kJ, with a median requested power per pulse of 37.5 W (range: 19–45 W) per tumor. All tumors were accessible to treatment, through dog positioning and the HIFU device articulation capabilities. Breathing motion under anesthesia was not a limitation (Figures 1).

**Figure 2.**
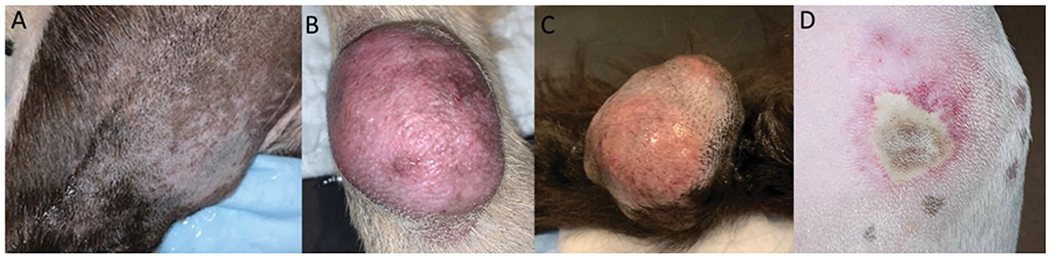
Thermal injury to the skin, 4–6 days post HIFU treatment. The haircoat over the tumor site was removed immediately before the treatment. (A) No evidence of thermal injury. (B) Second degree burn. (C) Third degree burn. (D) Fourth degree burn.

Adverse events attributed to the HIFU treatment included: 5 full thickness burns (3rd degree burn), 1 deep partial thickness burn (2nd degree), 1 superficial partial thickness burn (2nd degree) (Table 3), and 1 degranulation event in a patient diagnosed with a mast cell tumor. Third degree burns were visible almost immediately following HIFU treatment, whereas less severe burns became apparent 1–4 days post treatment. None of the patients required aggressive pain management or surgery to address the cutaneous thermal injuries, as patients did not exhibit obvious clinical signs or alterations to vital parameters consistent with discomfort, nor affect patient quality of life. The degranulation event was restricted to the tumor site and was addressed with the planned tumor surgical resection. Thermal injuries to skin were observed more frequently in patients with haircoats that were more challenging to remove completely (Figures 2).

Two patients developed complications not directly attributed to HIFU treatment. One patient diagnosed with osteosarcoma, developed a grade IV anemia following surgical tumor resection *via* hemipelvectomy, 4 days post HIFU treatment. The patient’s Packed Cell Volume (PCV) prior to surgery was unchanged when compared to the PCV measurement obtained the day of HIFU. The severe anemia that developed 24 h following surgery was consistent with anemia of chronic disease, blood loss anemia, and intravascular hemolysis. The dog subsequently developed acute kidney injury and was humanely euthanized. Unfortunately necropsy was declined by the owner, prohibiting a more definitive assessment of the complication pathogenesis. The second dog was diagnosed with a large soft tissue sarcoma localized over the left carpus. The dog received wide surgical excision followed by open wound management in order to spare the limb. The patient self-traumatized the wound bed post-surgery on multiple occasions, which resulted in infection and delayed wound healing (Figures 1 and 2).

### HIFU treatment efficacy

**Figure 3.**
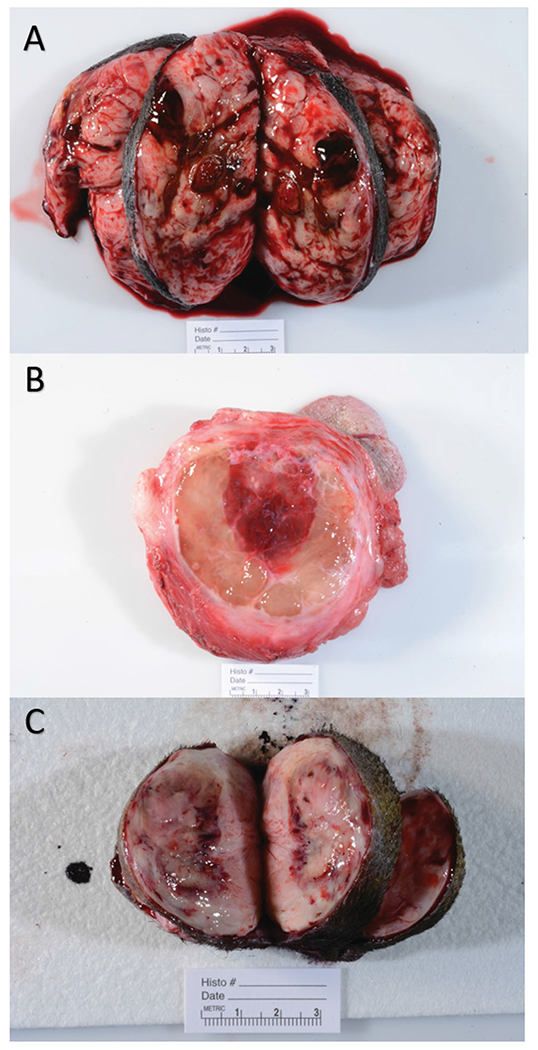
Gross images of 3 different representative treated tumor samples. Grossly, treatment areas were generally characterized by discrete foci of hemorrhage and tissue softening as seen in images (A and B). In some samples, treatment areas were less discrete and characterized by pallor with a rim of hemorrhage as seen in image (C).

Following surgery, samples were grossly evaluated for identification of ablated tissue, *via* use of anatomical landmarks (cutaneous and other anatomical landmarks such as hairless area of skin, permanent ink marked site of treatment, and pictures of the treated site). Tumor ablation lesions were identified grossly as focal regions of tissue softening and/or hemorrhage. These lesions varied but were generally sharply demarcated from the surrounding tumor tissue (Figure 3). In some cases, redness, edema, and/or ulceration of the skin overlying the treatment area was observed. All tissues were grossly reviewed and histopathologically assessed by a single board-certified pathologist (SCO). A combination of radial and tangential sections were obtained from the margins of the tumor, regions of the tumor surrounding the ablation zone, and the transition zone (interface of treated and untreated tumor).

Histopathology performed on tumors resected 4–6 days post treatment confirmed effective partial tumor ablation as defined by the presence of a sharply demarcated region of coagulative necrosis within the target site, in the 18 dogs treated according to protocol.

**Figure 4.**
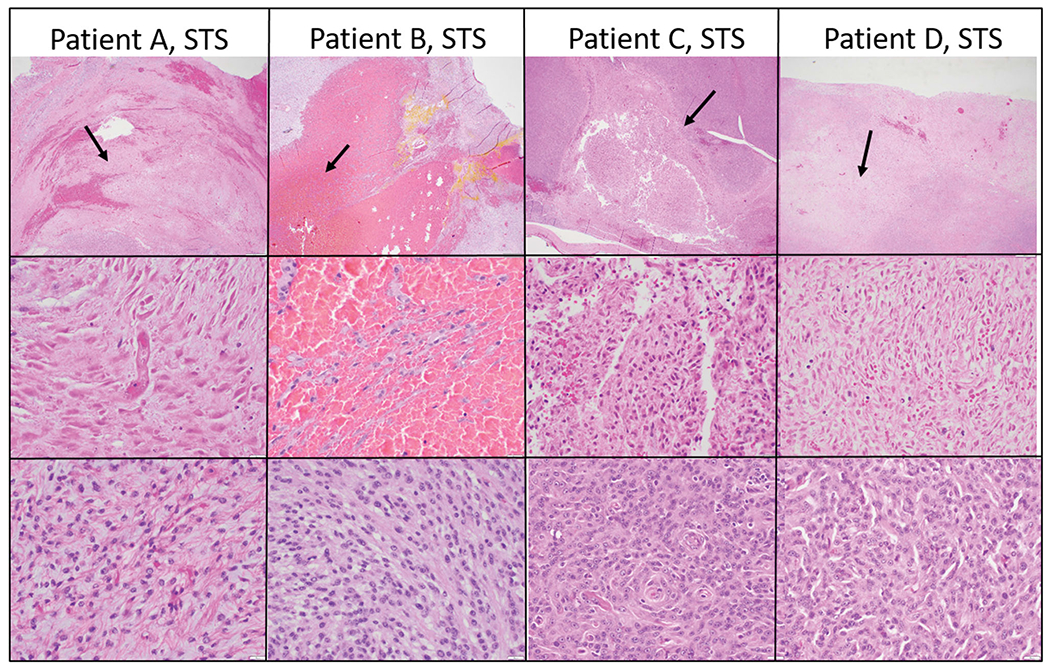
Select representative histopathology of patient tumors. This Figure illustrates the treated tumor at low magnification (top row), and high magnification (middle row) of four selected patients with soft tissue sarcomas that were treated with HIFU. Though treatment areas were somewhat variable, the most consistent finding is a relatively discrete focus of tumor necrosis (arrows) with hemorrhage at low magnification. In some patients (Patient B), hemorrhage and individualization of tumor cells were the most prominent feature. At higher magnification (middle row), necrotic cells exhibit coagulative necrosis characterized by individualization of cells, angular cell borders, increased ‘pinkness’ of the cytoplasm (hypereosinophilia), and dark nuclei or decrease in nuclear visibility. The bottom row illustrates what intact tumor cells look like for that patient in untreated areas of the tumor. Images taken at 2x (top row) or 40x (middle and bottom rows), H&E.

The ablated lesions were microscopically characterized by coagulative necrosis and hemorrhage (Figure 4). Necrotic cells were visible as ghost cells with angular cell borders, changes in tinctorial properties (hypereosinophilia), and condensed to barely visible nuclei. In cases where collagenous stroma was prominent, there was hyalinization of the stroma as well. Treatment areas exhibited fibrinoid necrosis of vessels and fibrin thrombi (Figure 4, middle row, patient 1). Of the treated tumor types, the soft tissue sarcomas exhibited the most consistent histologic changes. In other tumor types, the presence of hemorrhage was consistent, but the degree of necrosis was more variable and less discrete.

### Immunologic reaction to HIFU ablation

**Figure 5.**
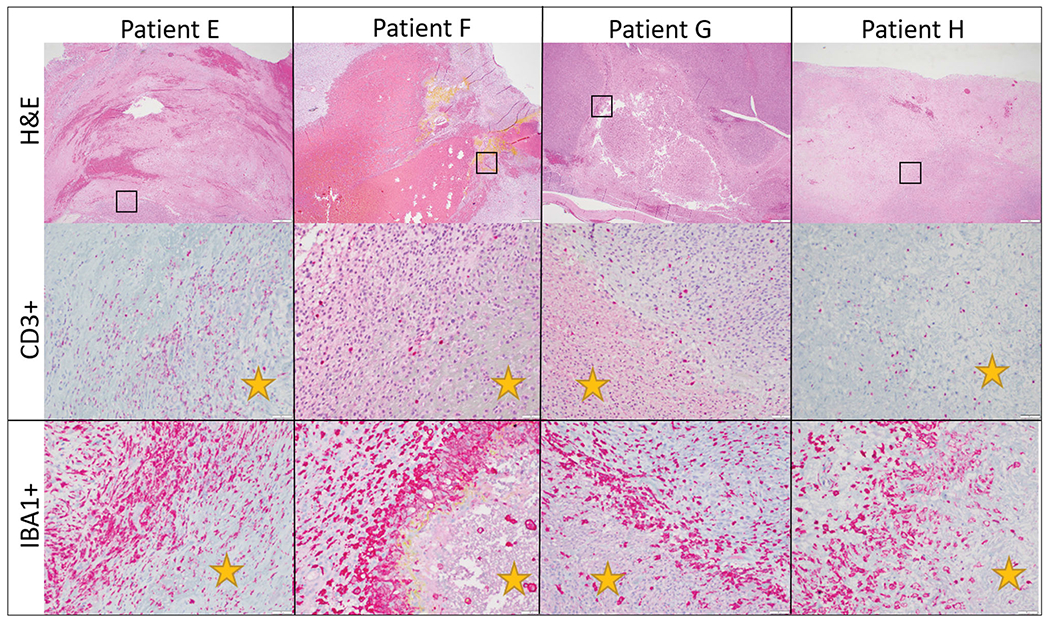
Representative immunohistochemistry images from patients treated in our HIFU study. The top row illustrates CD3 immunohistochemistry for dogs diagnosed with soft tissue sarcomas that were treated with HIFU while the bottom row illustrates IBA-1 immunohistochemistry for the same patients. Positive cells are stained red. CD3+ cells are present throughout the untreated tumor cells as well as at the interface and within the treated section. There is not a definitive or consistence difference in the number of CD3+ cells at the treatment interface. IBA1+ cells, however, consistently were high in numbers at the treatment interface though were also present within untreated and treated tumor as well. Treated tumor is marked with a yellow star. Images taken at 20x, DAB counterstain.

Following tumor sectioning as described previously, immunohistochemistry for CD3, CD79a, and IBA-1 was performed (Figure 5). IBA-1 positive cells were common throughout the neoplasms and were arranged in an individually scattered pattern amongst neoplastic cells. Numbers of IBA-1 positive cells were most intense at the ablated/non-ablated tumor tissue interface (transition zone), where necrotic cells abutted intact cells. Scattered, individual IBA-1 positive cells were also common in the treated areas in variable numbers. CD3 positive cells were higher in numbers throughout the untreated portions of the neoplasm. The number of CD3 positive cells appeared markedly increased in tumor margins and around small vasculature, in tumors with prominent lymphoid nodules. CD79a positive cells were variably present throughout all samples, but in low numbers. CD79a positive cells were rare at the treatment interface and not identified within the treated tissue. Collectively, the results indicate intra-tumor infiltration of IBA-1 positive cells at the ablated/non-ablated tumor interface and CD3 positive cells are concentrated in the tumor margins and vasculature of tumor that had preexisting intra-tumoral lymphoid follicles present.

Gene expression analysis identified 28 genes with statistically significant differential expression post-HIFU treatment, with 2 genes being downregulated and 26 genes upregulated (Table 4). Of the downregulated genes, PLAUR encodes the receptor for urokinase plasminogen activator, while VEGFA encodes for the vascular endothelial growth factor A, an essential factor for angiogenesis. The upregulated genes are associated with T-cell receptor signaling, B and T lymphocyte survival, NK maturation, chemotaxis promotion, and adhesion. The gene expression patterns are consistent with a proinflammatory microenvironment, characterized by increased signaling through the NF-κB and Wnt signaling pathways. Together, these data are consistent with augmented anti-tumor immune system signaling mediated, in part, by increased B- and T-cell activation.

### Clinical outcomes

**Figure 6.**
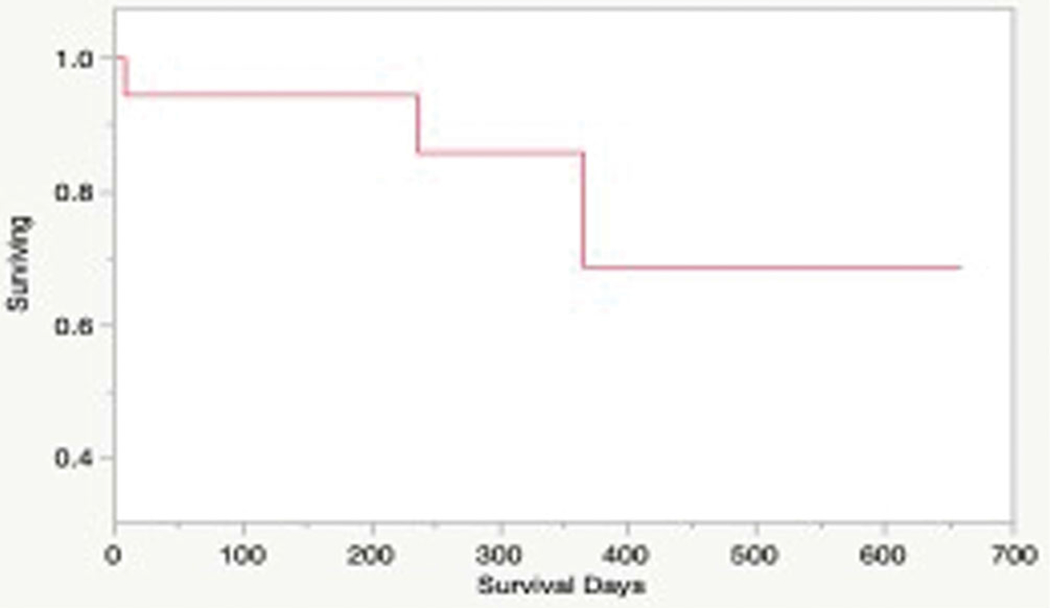
Kaplan-Meier survival estimate for dogs in the study treated with HIFU and tumor was resected (*N* = 15). The median overall survival time for the study population was not reached.

Neither the median survival time nor median time to progression was reached for patients in this study (Figure 6). 3/18 dogs treated according to protocol died during the study period, while the remaining 15 were censored from survival analysis, as they were alive without any evidence of disease at the time of manuscript preparation. Local tumor recurrence was confirmed in 1 patient during the study period, and suspected in another. Both patients had a history of incompletely excised soft tissue sarcomas and did not pursue adjuvant therapy. Another dog was euthanized due to development of metastatic gastric adenocarcinoma, unrelated to the original tumor treated with HIFU.

## Discussion

We report the results of the first prospective pilot study of HIFU for the treatment of solid tumors in dogs. Our results indicate that HIFU can be effectively delivered to canine subcutaneous solid tumors to result in complete thermal ablation of the targeted tissue. We characterized the short-term adverse event profile of delivering HIFU to canine subcutaneous tumors, and described the occurrence of thermal injury to the skin overlying the tumors. The presence of thermal damage, while not significantly affecting the quality of life of dogs in this study, is an important parameter to consider in future HIFU applications, as it developed despite the active cooling of the skin during the treatment. Additional work to optimize treatment protocols that mitigate the risks of thermal injury are needed, as the number of patients experiencing 3rd degree burns was higher than anticipated. Fortunately, none of the dogs in this study required surgery or pain/wound management to address these thermal complications.

When we investigated the effects of the HIFU in the tumor microenvironment, we observed a zone of viable tumor tissue around the ablated tumor that was characterized by infiltration of IBA-1 positive cells, that were morphologically and immunohistochemically consistent with macrophages. This finding supports the need for additional studies to further investigate the polarization or reprogramming of these highly plastic and heterogenous cells both before and after treatment. The previous designation of type I and type II macrophages may now be obsolete with newly identified subtypes described in the literature over the last decade, all playing different roles, while still maintaining various overlapping functions in certain cellular/disease contexts. A combination of multiplexed IHC allowing spatial assessment of the various subtypes as well as gene expression analysis following isolation of the cells would be the most fruitful in elucidating the function and identity of these cells. Unfortunately, specific markers and transcriptional programs for the various subsets of macrophages in canines are not well characterized.

Similarly, additional work to characterize the tumor infiltrating T-cell lymphocyte population seen in higher numbers throughout the untreated portions of the neoplasm and increased along the margins and small vessels. Clarifying the specific subtypes, differentiation status, and associated functions before and after treatment would allow for a better understanding of the impact of HIFU on the tumor microenvironment. Gene expression analysis using RNA isolated from the cells at the transition zone (zone between ablated and non-ablated tumor tissue) indicated that HIFU-treated soft tissue sarcomas may result in increased gene expression of genes associated with T-cell activation compared to pretreatment controls, and supports the need for further investigation and corroboration of these findings.

Although survival was not a primary endpoint of this study, it is important to note that partial tumor ablation with HIFU did not appear to adversely affect the median survival time for the current study population within the time period of follow-up. The median survival time was not reached, as the majority of dogs in our study population were diagnosed with tumors having a low risk for metastasis. The rate of recurrence and survival times are in agreement with previously published studies for patients receiving surgery in the literature [39,40].

The limitations of our study are consistent with those commonly encountered in small feasibility studies of novel therapeutics in pet dogs with cancer. While we attempted to draw our comparisons on the immunologic response of the host to the HIFU treatment using paired pre and post treatment samples, tumor heterogeneity may have a significant effect on the results of our study. Furthermore, the current classification of STS in the dog does not allow for stratification of different STS subtypes, thus limiting the potential identification of STS subtypes that may be more susceptible to ablation-induced tumor microenvironment manipulation.

While the thermal effects of HIFU in tumor tissue are well characterized, the effects of HIFU on the tumor microenvironment are not well understood, especially in the context of different tumor types. Our work demonstrated the safety and feasibility of treating tumors in close proximity to the skin with HIFU, in dogs diagnosed with spontaneously occurring cancer. As the use of HIFU is expanding in people, it becomes important to understand the limitations and adverse risk for treating superficial tumors over haired skin. Using pet dogs diagnosed with cancer as a model, represents an attractive opportunity to study the effects of thermal tumor ablation in an immunocompetent host, and provide insight in to the complex response of the tumor microenvironment to the treatment. This approach provides both the opportunity and rationale for mechanistic rodent studies to validate our results, and supports the use of pet dogs as a synergistic model in the design of HIFU ablative trials for people diagnosed with solid cancer.

## Figures and Tables

**Table 1.** Inclusion/exclusion criteria.

Inclusion criteria	Exclusion criteria
Subcutaneous solid tumor = or > 1cm	Subcutaneous solid tumor <1cm
No significant biochemical abnormalities concerning for inadequate organ function	Significant biochemical abnormalities concerning for moderate to severe organ dysfunction
Karnofsky performance score <2	Karnofsky performance score ≥ 2
No previous treatment or treatment with immunotherapy or anti-neoplastics > 3 weeks prior to enrollment	Treatment with immunotherapy or anti-neoplastics within previous 3 weeks
Expected survival > 6 weeks	Expected survival <6 weeks

**Table 2.** Patient characteristics of dogs enrolled in the study.

Breed	
Pure Breed	12 dogs (11 different breeds represented)
Mixed Breed	8 dogs
Sex	
Male	12 dogs
Female	8 dogs
Neuter Status	
Castrated	11 dogs
Spayed	7 dogs
Intact (M/F)	2 dogs (1 male and 1 female)
Age	
Years (median)	10.5 years (range 7–13 years)
Weight	
Kilograms (median)	30.95 kg (range 6.8kg–48.6kg)
Anatomical tumor location	
Extremities	15 dogs
Trunk	3 dogs
Tail	1 dog
Neck/cervical region	1 dog
Tumor histology	
Soft tissue sarcoma	15 dogs
Mast cell tumor	3 dogs
Osteosarcoma	1 dog
Thyroid carcinoma	1 dog

**Table 3.** Thermal Injuries (Burn scores) induced by HIFU at day of tumor surgical resection (day 4–6 post HIFU), adapted by Wohlsein et al.

No skin Burn	11
1st Degree Burn	1
2nd Degree Burn (superficial)	2
2nd Degree Burn (deep)	1
3rd Degree Burn	5
4th Degree Burn	0

**Table 4.** Genes with statistically significant expression changes post HIFU, in the Soft Tissue Sarcoma group.

Symbol	Description	Fold change	*p*-value
CD3E	CD3e molecule, epsilon (CD3-TCR complex)	11.3929	.011236
CD3D	CD3d molecule, delta (CD3-TCR complex)	10.925	.011583
DLA-DOB	major histocompatibility complex, class II, DO beta	9.56251	.007551
CCL21	chemokine (C-C motif) ligand 21	8.92264	.021212
TRBC		7.90738	.013974
CD22	CD22 molecule	7.8532	.013559
CD3G	CD3g molecule, gamma (CD3-TCR complex)	7.32257	.017454
TCF7	transcription factor 7 (T-cell specific, HMG-box)	6.69229	.015112
SLAMF6	SLAM family member 6	6.11109	.024896
CR1L		6.07894	.031854
SELE	selectin E	5.95124	.014139
IL2RG	interleukin 2 receptor, gamma	5.35498	.044532
TOX	thymocyte selection-associated high mobility group box	5.32352	.003619
LCK	lymphocyte-specific protein tyrosine kinase	5.12961	.014522
TNFSF11	tumor necrosis factor (ligand) superfamily, member 11	4.78822	.040326
CDH1	cadherin 1, type 1, E-cadherin (epithelial)	4.27929	.048927
TNFRSF13C	tumor necrosis factor receptor superfamily, member 13C	4.19875	.014028
CYFIP2	cytoplasmic FMR1 interacting protein 2	4.13925	.010465
S100A8	S100 calcium binding protein A8	3.64829	.037835
LOC100049001	tryptase	3.11392	.027965
ITGA4	integrin, alpha 4 (antigen CD49D, alpha 4 subunit of VLA-4 receptor)	2.92106	.009146
GH1	growth hormone	2.87671	.00263
LOC477699		2.53728	.045067
NT5E	5′-nucleotidase, ecto (CD73)	2.42019	.043786
KIT	v-kit Hardy-Zuckerman 4 feline sarcoma viral oncogene homolog	2.22728	.014551
ETS1	v-ets avian erythroblastosis virus E26 oncogene homolog 1	2.15832	.039782
VEGFA	vascular endothelial growth factor A	−3.06498	.010696
PLAUR	plasminogen activator, urokinase receptor	−3.71592	.045821

Mean fold change in gene expression following HIFU treatment compared with baseline, normalized using housekeeping controls is presented.

## Data Availability

Data were generated at the Virginia-Maryland College of Veterinary Medicine and are available from the corresponding author JC on request.
